# Supplementary material for: Nanoformulated Zoledronic Acid Boosts the Vδ2 T Cell Immunotherapeutic Potential in Colorectal Cancer
Source: Cancers (Basel). 2019 Dec 31;12(1):104. doi: 10.3390/cancers12010104 (PMC7017311; doi:10.3390/cancers12010104)
Supplement: Supplementary file 1 [file cancers-12-00104-s001.pdf]

Supplementary materials

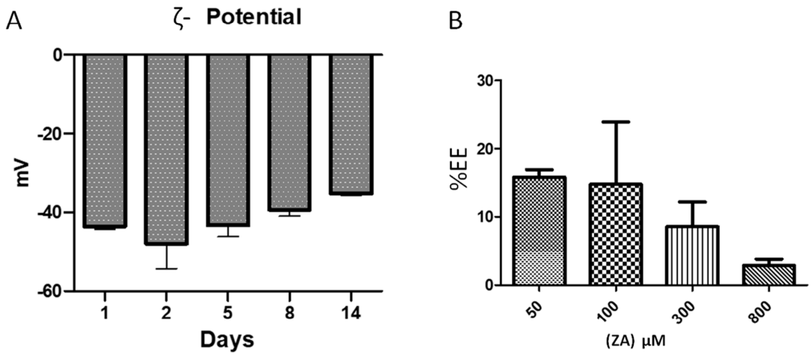

Supplemental Figure 1 (S1). ZA-SPNs ζ-potential over time and encapsulation efficiency.A. ZA-SPNs were monitored for their ζ-potential, expressed in mV, up to 14 days as a marker of nanoparticles stability**. B.** ZA-SPNs formulations, made out with different ZA input conditions, namely 50, 100, 300 and 800µg, were evaluated for their drug encapsulation efficiency (EE). Results are expressed as percentage of EE that represents the percentage in weight of the final ZA amount over the initial feeding amount.

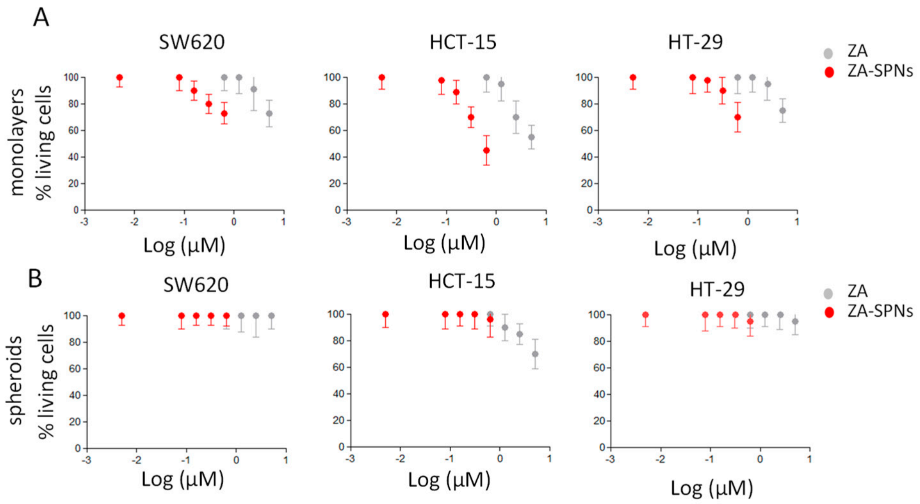

Supplemental Figure 2 (S2). Effect of ZA-SPNs and ZA on CRC viability. A. The CRC cell lines SW620, HCT-15, HT-29 growing adherent (monolayer) were incubated with ZA-SPNs (red) or with soluble ZA (gray) at different concentrations (Log µM), as indicated, for 48h, then viability was assessed by crystal violet assay. Data are expressed as percentage of living cells referred to cells cultured in medium alone and are the mean ± SD of 3 independent experiments of 6 different replicates for each condition. **B.** Spheroids of SW620, HCT-15, HT-29 were incubated with ZA-SPNs (red) or with soluble ZA (gray) at the indicated concentrations (Log µM) for 48h. Spheroid cell viability was assessed by a modification of crystal violet assay as described (43). Data, expressed as percentage of living cells referred to cells cultured in medium alone, are the mean ± SD of three independent experiments of 6 different replicates for each condition.

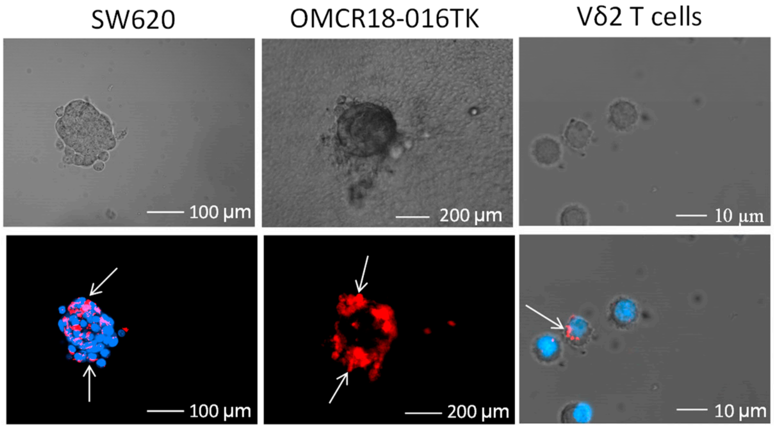

Supplemental Figure 3 (S3). ZA-SPNs are internalized by CRC spheroids and organoids. Confocal microscopy of SW620 spheroid, or organoid OMCR18-016TK, or Vδ2 T cells obtained from PBMC, as indicated, exposed to 0.05 µM Cy5-ZA-SPNs (red) for 24h. Spheroids or Vδ2 T cells were seeded onto glass slides, while organoids were analyzed in Geltrex domes. Samples were run under a FV500 confocal microscope and data analyzed with FluoView 4.3b software (Olympus). Images were taken in sequence mode and shown in pseudocolor.Upper pictures: bright field images. Lower pictures: dark field, left and central panels; merge bright and dark field, right panel. Spheroids (left) and Vδ2 T cells (right) were also stained with 20 nMSyto16 to identify nuclei (blue).Arrows indicate Cy5-ZA-SPNs.

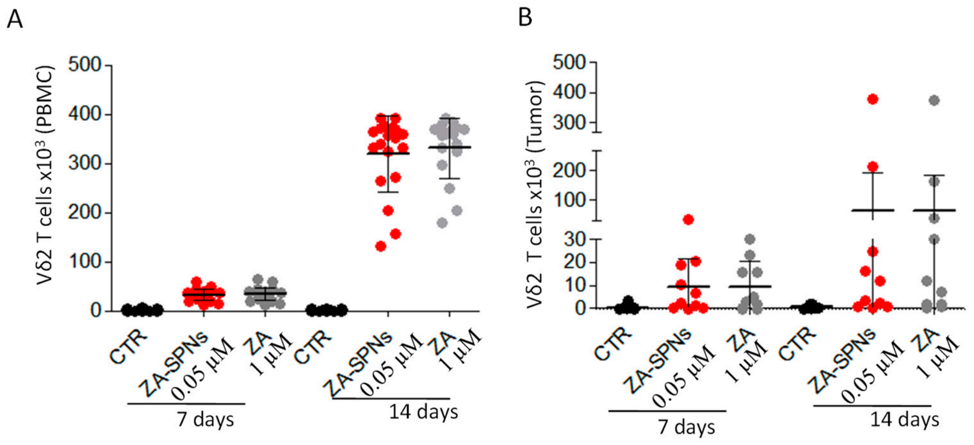

Supplemental Figure 4 (S4). ZA-SPNs trigger the expansion of CRC patients' Vδ2 T lymphocytes. A. PBMC from CRC patients were exposed to ZA-SPNs (0.05 µM) or soluble ZA (1.0 µM) for 24h and cultured with 10 ng/ml (30 IU/ml) IL-2. The percentage of Vδ2 T lymphocytes was evaluated by FACS analysis on day 7 and 14 and the absolute number referred to the total number of cells recovered. Mean ± SD of 20 patients. **B.** Tumor cell suspensions were cultured as in A, and Vδ2 T lymphocyte expansion was determined by FACS analysis on day 7 and 14. Results are expressed as absolute number of Vδ2 T lymphocytes referred to the total number of cells recovered; mean ± SD of 10 patients' specimens.

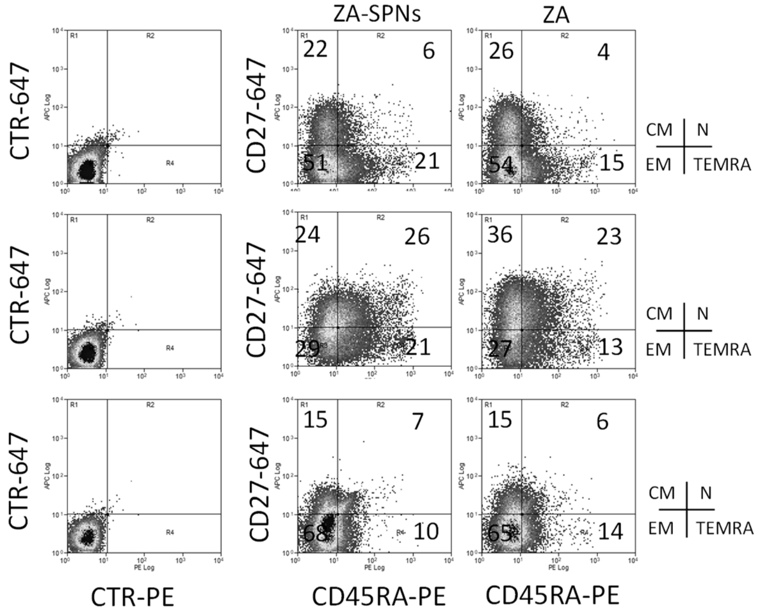

Supplemental Figure 5 (S5). Expression of CD45RA and CD27 in Vδ2 T lymphocytes after stimulation with ZA-SPNs. PBMC of three CRC patients were evaluated for the reactivity with anti-CD45RA and anti-CD27 mAbs upon stimulation with either 0.05μM ZA-SPNs (middle dot plots) or 1μM of soluble ZA (right dot plots) and culture for 21 days in the presence of IL-2. Cells were stained with the indicated mAbs followed by isotype specific GAM antiserum. Each dot plot is subdivided in four quadrants: CD45RA$^-$CD27$^-$ cells (effector memory cells, EM, lower left) CD45RA$^+$CD27$^-$ cells (terminal effector memory cells, TEMRA, lower right) CD45RA$^+$CD27$^+$ cells (naïve cells, N, upper right) and CD45RA$^-$CD27$^+$ cells (central memory cells, CM, upper left) to identify different subsets of Vδ2 T lymphocytes. The number in each quadrant indicates the percentage of the Vδ2 T lymphocytes with that phenotype. The dot plots on the left show the immunofluorescence staining with the isotype specific GAM without primary antibody on cell populations obtained with ZA-SPNs to set the background level of immunofluorescence. Results are expressed as Log red fluorescence intensity versus Log far red fluorescence intensity in arbitrary units.

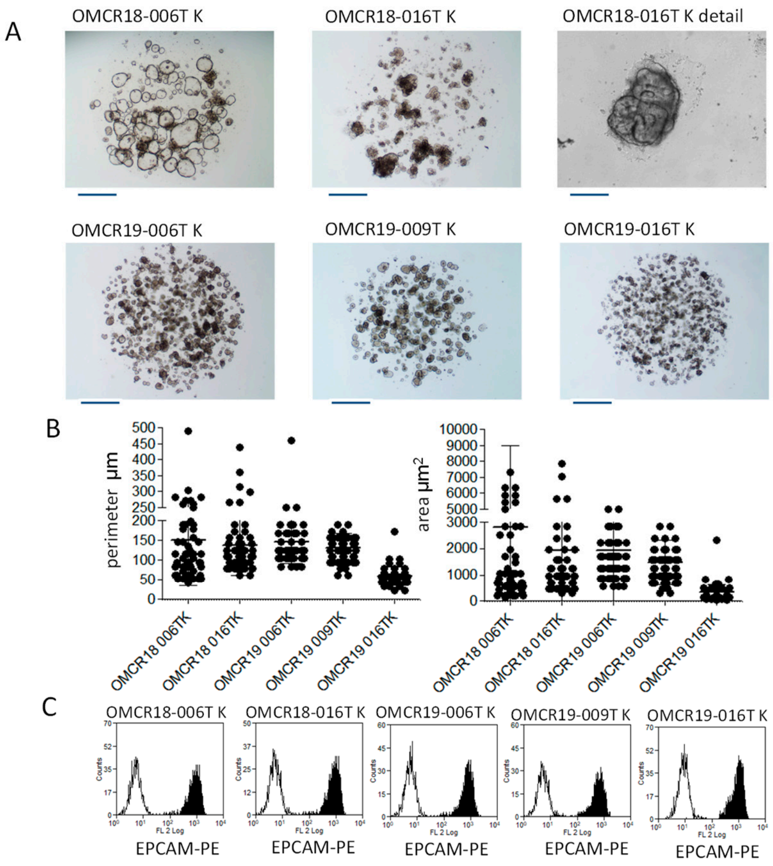

Supplemental Figure 6 (S6). Colon organoid characterization. A. Images of the primary CRC organoid cultures used in this study (Suppl.Table I). Single Geltrex domes (3µl), containing growing organoids in standard culture medium, were photographed by a Leica DM-LB2 microscope, equipped with a GX-CamU3-18 camera. 50x magnification. Bar: 1000µm. OMCR18-006TK shows a morphology typical of mucinous tumors (see Suppl.Table I). The upper right panel represents a detail of the OMCR18-016TK organoid culture in a different field. 200x magnification. Bar: 100µm. **B.** Perimeter and area of CRC organoids of the indicated patients determined using the CellR software (Olympus Biosystem).**C**. Expression of EPCAM (black histograms) on the organoids depicted in **A**, evaluated by indirect immunofluorescence with the specific mAb, followed by the PE-conjugated anti-isotype specific GAM antiserum, and FACS analysis. Empty histogram: PE-conjugated GAM alone.

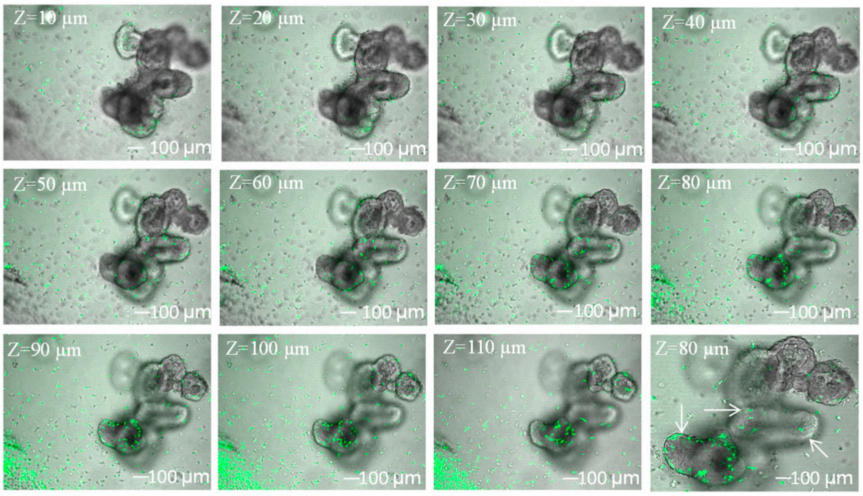

Supplemental Figure 7 (S7). ZA-SPNs-expanded Vδ2 T cells can infiltrate colon organoids. The CRC OMCR18-016TK organoids, cultured in a Geltrex dome, were challenged with ZA-SPNs-expanded Vδ2 T cells labeled with CFSE (green) and the initial infiltration of Vδ2 T cells of the organoid was assessed at 48h. Samples were run under a FV500 confocal microscope and analyzed with FluoView 4.3b software (Olympus). Images were taken in sequence mode and shown in pseudocolor. The panels represent images taken at different Z points (see the Z position on the upper left corner of each panel) with a 20x objective. The last image on the lower right hand panel is an enlargement of Z=80μm point and the arrows indicate the Vδ2 T cells that appear with an elongated shape, like intra-epithelial cells in the crypt-like structure of organoids.

Supplemental Table 1. Pathology features of CRC patients from which tumor cell suspensions and/or organoids have been derived.

**Patient ID**[a]	**Sex**	**Age**	**Localization**[b]	**Stage UICC**[c]	**Stage Dukes**[c]	**MS status**[d]
OMCR18-053T K[e]	M	72	Right	pT3/G2/N0	B2	MSS
OMCR18-059T K[e]	F	79	Rectum	pT2/G2/N0	B1	MSS
OMCR18-060T K[e]	M	79	Rectum	pT3/G2/N0	B2	MSS
OMCR18-061T K[e]	F	62	Right	pT2/G3/N1b	C1	MSS
OMCR19-005T K[e]	F	81	Rectum	pT3/G2/N0	B2	MSS
OMCR19-007T K[e]	F	68	Rectum	pT1/G2/N0	A	MSS
OMCR19-013T K[e]	M	84	Right	pT3/G2/N0	B2	MSS

OMCR19-014T K[e]	F	67	Right	pT3/G2/N0	B2	MSI
OMCR19-015T K[e]	M	79	Sigma	pT3/G2/N1b	C2	MSS
OMCR18-006T K[f]	M	79	Right	pT3/G2/N0	B2	MSS (mucinous)
OMCR18-016T K[f]	M	43	Transverse	pT3/G2/N0	B2	MSI
OMCR19-006T K[f]	M	68	Rectum	pT2/G2/N0	B1	MSS
OMCR19-009T K[e, f]	F	82	Right	pT4a /G3/N2a	C2	MSS
OMCR19-016T K[f]	M	77	Right	pT3/G3/N0	B2	MSI

[a] Patients' identification number (ID). [b] Localization of tumors was determined by the surgery staff of the Oncological Surgery Unit: see Supplementary Table I.[c] The tumor stage was determined according to UICC or Dukes classification modified by Aster and Coller [60]. [d] Microsatellite status was analyzed from the Pathology Unit of the IRCCS Ospedale Policlinico San Martino. [e]Cell suspensions were isolated from the surgical specimens of the indicated patients, and used as described in Materials and Methods. [f] Organoids were obtained from the indicated patients and used (Materials and Methods) within the fifth passage of culture.
